# Supplementary material for: A Cross-Sectional Survey on the Clinical Management of Emergence Delirium in Adults: Knowledge, Attitudes, and Practice in Mainland China
Source: Brain Sci. 2022 Jul 26;12(8):989. doi: 10.3390/brainsci12080989 (PMC9332432; doi:10.3390/brainsci12080989)
Supplement: Supplementary file 1 [file brainsci-12-00989-s001.zip › brainsci-1793266-supplementary.pdf]

Table S1. The completed version of the questionnaire.

A cross-sectional survey on the clinical management of emergence delirium in adults: knowledge, attitude, and practice in mainland China

1. What is your post of duty in the hospital?
□ Anesthesiologist
□ Anesthesia nurse
2. How many years have you been working in clinical anesthesia?
_________ years
3. How many beds in total in your hospital? ______beds
How many surgeries per year in your hospital? ______
How many cases of general anesthesia per year in your hospital? _______
4. Do you think Emergence Delirium (ED) is an important clinical issue?
□ Very important
□ Quite important
□ Not very important
□ Not important
5. Do you think the management of ED is an important clinical issue?
□ Very important
□ Important
□ Not important
6. What do you think could be the risk factors for ED? (Multiple choice)
□ Advanced age
□ Comorbidities
□ ASA status
□ Preoperative fluid fasting
□ Hyponatremia or hypernatremia
□ Alcohol-related disorders
□ Anticholinergic drug
□ Site of surgery
□ Intraoperative bleeding
□ Duration of surgery
□ Postoperative pain
□ Catheter-related discomfort
If your options were not included in the abovementioned answers, please list them in the blank_______________
7. What do you think could be the preventions for ED? (Multiple choice)
□ Fast-track surgery
□ No premedication with benzodiazepines
□ Preoperative risk factors evaluation
□ Monitor depth of anesthesia
□ Adequate perioperative pain management
□ Promptly diagnosing of delirium
If your options were not included in the abovementioned answers, please list them in the blank_______________
8. Do you think it is necessary to monitor the DOA?
□ Routine monitor
□ Only routine in older adult patients
□ Only routine in high-risk patients
□ Never monitor
If you have chose the first three options, please list the monitor device in your clinical practice for DOA.
□ BIS
□ Sedline
□ EEG
□ rSO2
□ TCD
□ any device more than abovementioned, please list in the blank_________

9. Do you routinely assess ED?
□ Routine
□ Only routine in older adult patients
□ Only routine in high-risk patients
□ Never
10. If you assess ED, please choose the tools you use.
□ Clinical symptoms
□ Confusion Assessment Method (CAM)
□ Bedside Confusion Scale (BCS)
□ Cognitive Confusion Scale (CTD)
□ Clinical Global Impression Scale Delirium (CGID)
any tool more than abovementioned, please list in the blank_________
11. What do you think it should be evaluated for ED?
□ Diagnosis of delirium
□ Classification of delirium
□ Severity of delirium
□ All above
12. Have you ever been with ED?
□ Yes
□ No
If the answer of Q12 is Yes, what was the classification of the delirium?
□ Hyperactive
□ Hypoactive
□ Mix
If the answer of Q12 is Yes, what was the location when ED happened?
□ In the OR
□ In PACU
□ Both OR and PACU
How do you think the role of anesthesia nurse during the management of ED?
□ Very important
□ Important
□ Not important
13. What kind of treatment(s) do you think is proper for ED? (Multiple choice)
□ Nothing
□ Shout to inhibit aggressive behavior
□ Restraint
□ Artery blood gas analysis
□ Sedation
□ Pain management
If your options were not included in the abovementioned answers, please list them in the blank_______________
14. What do you think is the most effective medication for ED?
□ Intravenous analgesia
□ Propofol
□ Midazolam
□ Dexamedetomidine
□ Haloperidol
□ Droperidol
If your options were not included in the abovementioned answers, please list them in the blank_______________
